# The Role of Disability in the Relationship Between Mental Health and Bullying: A Focused, Systematic Review of Longitudinal Studies

**DOI:** 10.1007/s10578-022-01457-x

**Published:** 2022-10-23

**Authors:** Lilly Augustine, Ylva Bjereld, Russell Turner

**Affiliations:** 1 CHILD, School for Learning and Communication, Jönköping University, Jönköping, Sweden; 2 Department of Behavioural Sciences and Learning (IBL), Linköping University, Linköping, Sweden; 3 Department of Social Work, University of Gothenburg, Gothenburg, Sweden

**Keywords:** Bullying, Literature review, Mental health problems, Disability, Symptoms

## Abstract

Having both a disability and being bullied increases the risk of later mental health issues. Children with disabilities are at greater risk of being bullied and therefore at greater risk of adverse mental health outcomes. We conducted a limited systematic review of longitudinal studies focusing on the role of disability in relation to bullying and mental health problems. Twelve studies with an initial measure of mental health or disorder, measured no later than 10 years of age, were found. Ten of these twelve studies suggested that having a disability before victimisation increased the impact of mental health problems measured after bullying experiences. The conclusion is that children with a disability, such as behavioural problems, have an increased risk of later mental health problems through bullying victimization. Children with two risk factors had significantly worse mental health outcomes. These additional mental health problems may be alleviated through reduced bullying victimisation.

## Introduction

Being bullied is harmful with negative consequences for the victim. It is one of the main reasons why adolescents feel unsafe in school [1] and a significant environmental trauma associated with mental health problems (MHP) [2]. Bullying constitutes a severe form of harassment that is systematic and repetitive, where the victim loses power over the aggressor [3, 4]. Children with disabilities, both with and without a diagnosis, are at a higher risk of being bullied [5–7].

A transactional model can be used to theoretically explain the relationship between disability, bullying, and MHP. According to a transactional model [8], children’s development is shaped in a reciprocal interaction with the immediate environment. Further, children’s previous experiences affect their interpretations of new interactions, and certain traits will be strengthened or weakened in the transactional process [8]. While a disability can be thought of as an impairment related to the individual, a fuller account of disability can be viewed as the *impact* of a disability that is created within the transactions between the individual and their environment. Similarly, Bronfenbrenner’s bioecological model also draws attention to the importance of interaction between the person, the context, and the processes between them, i.e., the interaction will, over time, drive development [9]. Development can be seen as the product of a reciprocal interaction between a person and their environment, i.e., in the proximal process [10]. In such a model, bullying is a powerful contextual element that repeats over time, forming part of a proximal process. Bullying victimisation is likely to affect development substantially, given that repetition is a part of the definition of bullying. Moreover, the loss of power experienced by the victim is expected to impact or restrict a child’s interactions with the world, and affect how the child then interprets new social situations. Especially where bullying is chronic, maintained over several years or even school transitions [11], this will arguably affect school and social functioning and have longitudinal effects on health, especially psychological factors, such as mood, stress, and concentration. Previous research has shown that bullying victimisation increases the risk for MHP [12–15], social problems [16] and physical health issues [17]. There is also a risk of these issues continuing into adulthood [15].

The WHO definition of health is complete physical, mental, and social wellbeing and not merely the absence of disease or infirmity [18]. This definition suggests that factors that affect wellbeing in the short or long term, such as bullying, are problematic and detrimental to health [2]. It also indicates that disability does not reduce wellbeing but can be a risk factor for developing adverse health due to environmental barriers or responses [19]. In the International Classification of Functioning, health, and disability (ICF) [20], a shift toward a biopsychosocial model of disability and health has emphasised the role of environmental factors in creating disability. In other words, how impairments impacts, through environmental factors, to shape disability. However, the ICF is a complement to the global standard for diagnostic health information International Classification of Diseases (ICD), which focuses on classifying functioning and disability via diagnoses. Hence, the ICF defines disability differently compared to the ICD.

In the current study, we adopt the view, more closely aligned to the ICF, that disability involves dysfunction related to impairments, activity limitations, or restrictions on participation in ordinary life. In this view, disability and functioning are the outcomes of the *interaction* between health conditions, including bodily functions and structure, and contextual factors. A person’s activity and participation in everyday tasks become vital aspects of this interaction. Barriers to involvement in ordinary life situations can limit functioning, as well as health and well-being. Based on the ICF’s definition of disability, symptoms visible in life situations can be seen as disabling, without necessarily being a confirmed diagnosis. Such symptoms can reduce physical and social functioning, thus impairing the ability to adjust to or positively grow with one’s surroundings.

The benefit of using the ICF’s definition of disability is its breadth of scope. For example, it includes children who have impairments relating to mental functioning, such as difficulties with attention or the regulation of emotions. It also includes children who have restricted physical activity that can affect their capacity to handle general tasks and demands. Within a transactional process, both these children will have visible symptoms within a context, independent of a medical diagnosis or not. Hence, this definition of disability can include those with a diagnosed disorder as well as those children who display symptoms but do not have a confirmed diagnosis.

### The Relationship Between Disability, Symptoms of Mental Health Problems, and Bullying Victimisation

Becoming a victim of bullying is not just a random event [21]. Bullying victimisation can be stable over time and is associated with symptoms of MHP [11], and this association can go both ways [22]. There are also indications that early symptoms of MHP are one of the risk factors for being bullied. At the same time, being bullied can contribute to adjustment problems increasing the likelihood of MHP [15, 16]. Children with disabilities [5], as well as children with symptoms of internalising [21] or externalising behaviours [6, 22], have an increased risk of being bullied. A systematic review showed that children with chronic physical or sensory disabilities had a higher risk of being bullied and a higher risk of bullying others, even if the risk for bullying others was lower than the risk of being bullied [7]. Difficulties with social relations may be part of the reason why children with disabilities are more prone to being bullied, as bullies often choose victims rejected by peers [23]. Other systematic reviews confirm this and found that children with autism or intellectual disabilities had an increased risk of being bullied [24, 25]. The reasons for these findings might be many. Arsenault, Bowes and Shakoor [6] argued that children’s behavioural problems might send signals that they are easy prey or that they already have lower status. Children with aggressive tendencies may trigger hostility in others. This transactional pattern corresponds well with a bioecological framework, i.e., that there is a need to be able to adapt to the demands of the surrounding social environment [9]. Adaptation, however, is the product of both personal functioning and contextual factors that interact to push development in a specific direction. Therefore, having a disability or lower psychological functioning and being exposed to bullying arguably, increase the risk of a negative adaptation and, thus, later psychological problems.

Thus, it can be concluded that having a disability or being bullied increases the risk for a later MHP [5, 6, 15, 21]. However, it is less clear what the longitudinal associations are between diagnosed disorders or symptoms of MHP in childhood (e.g., disability), bullying victimisation, and later MHP during adolescence. Do children with early (i.e., preadolescent) symptoms of MHP or a disorder have a higher risk of being bullied and worse mental health outcomes in later adolescence when bullied? While many individual studies address this question, there is no review of the specific topic. Thus, the current study aims to review existing longitudinal research on the relationship between bullying and mental health among children with disabilities. We hypothesise that children with disabilities will have a higher risk of developing MHP if they have been bullied than children who have not been bullied or only have a disability (i.e., not bullied). Further, we hypothesise that children with disabilities, either as a diagnosed disorder or as symptoms of MHP in childhood, have a higher risk of developing MHP in adolescence if they have been bullied, compared to: (a) children with disabilities who have not been bullied,and (b) bullied children without a disability.

## Method

When investigating MHP outcomes in studies of children with disabilities/symptoms of MHP and bullying, the measurement of early symptoms of MHP needs to occur before the measurement of bullying. Similarly, the measurement of bullying needs to occur before measuring later MPH outcomes. The measurement thus needs to be in the order of: (1). disability, either as a disorder or as early symptoms of MHP; (2). Bullying; and (3). the relation to later MHP in longitudinal studies. As bullying is more prevalent in early adolescence [26, 27], studies with the first point of measurement in adolescence might miss essential aspects of the relation between early, i.e., childhood, symptoms and later mental health outcomes concerning bullying, for example, in children exposed to chronic bullying. Therefore, the current review only includes studies with initial measurements before 10 years of age to isolate the effect of bullying on functioning or on later MHP.

This study is a limited systematic review. The literature searches for the review were conducted in multiple search engines in August 2019 and again in August 2020 to include any recently published studies. Databases used were CINAHL, ERIC, MEDLINE, Psych Info, Sociological abstracts, PubMed, Swepub, open grey, OECD library, and Scopus. Primarily thesaurus terms were used, though these differed somewhat depending on databases. All terms were related to MHP for children with disabilities, bullying or peer victimisation, and comparisons with other children [for specific search strings used, see supplement (1)]. The thesaurus terms used reflected our definitions of participants, exposure, comparison-group, and outcome (PECO). Table 1 shows the PECO criteria.

**Table 1:** PECO and inclusion and exclusion criteria

	Inclusion	Exclusion
Participants	Children with disabilities, disorders, impairments, chronic conditions, or reduced functioning	Studies with the initial measurement at 10 years of age or later without a subgroup possible to extract
Exposure	Bullying Peer-victimization	Studies with other aspects of victimisation or traumatisation included in the measurement Bullying in other types of context than school, such as institutions Bullying is measured concurrently with MHP
Comparison	Children with disabilities who are not bulli ned or bullied children without disabilities	
Outcome	MHP, internalising or externalising problems	No outcome of mental health Outcomes are not measured after exposure Exposure is measured retrospectively

### Procedure

**Fig. 1:**
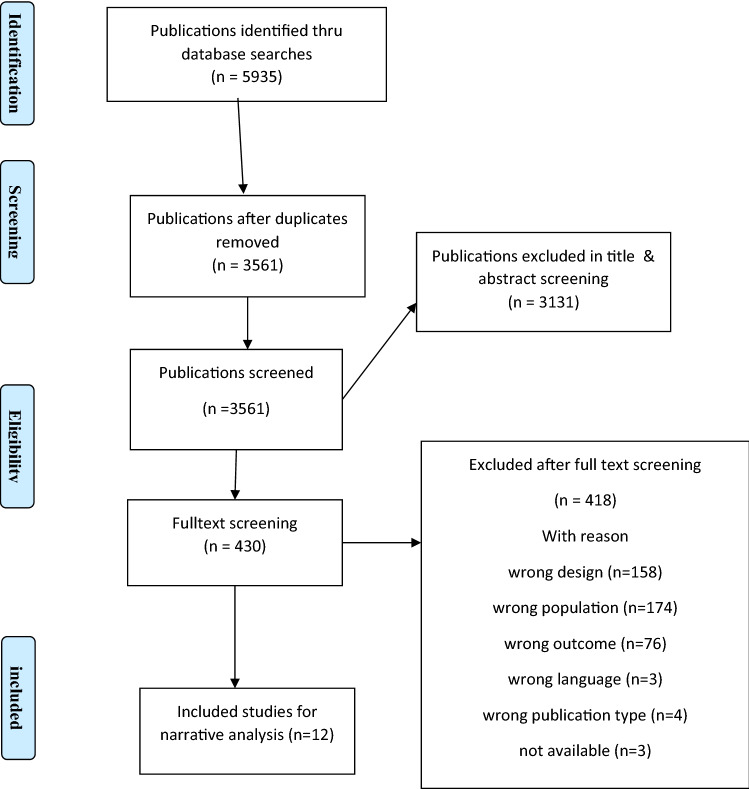
Flow chart of the screening process

Search strings were created for each database based on the study’s aim and PECO to identify studies eligible for inclusion. Results across the different databases were merged, first in Endnote for deduplication. After that, the remaining articles were imported into the Rayyan QCRI software tool [28], and in Rayyan, additional duplicates were identified and removed. The two reviewers, authors one and two, screened 3432 titles and abstracts for eligibility. The inter-rater agreement between reviewers was 90.4%. The articles reviewers did not agree upon were included in the full-text screening. The first reviewer then full-text screened the remaining papers (k = 430). The second reviewer screened a random sample of these (k = 108). The interrater agreement for the full-text screening was 85%. All screening was carried out independently by the reviewers. The articles disagreed upon were discussed, and then a second screening was performed. See Fig. 1 for the screening process [29].

A total of 12 studies were included based on the screening process. Quality assessment was conducted using The Critical Appraisal Skills Programme (CASP) for cohort studies [30]. Then, a qualitative synthesis was performed and reported narratively. Based on the CASP, all articles were assessed based on ten questions, coded from 0 to 2 points (maximum 20 points). Higher quality ratings relate mainly to solid methodology because the quality assessment in CASP focuses on design, recruitment, analysis, and generalizability. Many of the included studies were considered to have good quality, as they were part of more extensive programs with straightforward designs and follow-ups.

In Table 5 in Appendix, all articles are presented. Each publication has been assigned a unique number used in the tables, and this corresponds to the number in the appendix related to each reference.

## Results

This review investigated the longitudinal associations between disability/early symptoms of MHP, bullying victimization, and later additional MHP. Most of the 12 included studies used a measure of symptoms of MHP at baseline (k = 8). These studies had a symptom-driven perspective, which is more in line with the ICF definition of disability that we follow, whereby psychological symptoms in early life were related, via subsequent bullying, to later mental health problems. All but one of these studies were rated as having medium or high quality and had long follow-up periods. Three of the studies used register data as outcomes. Five of the twelve included studies used a diagnosis of disability or disorder as its baseline measure.

### Studies Using Measures of Symptoms of MHP in Childhood

Of the identified studies that used symptoms of MHP in childhood as a measure of disability (k = 8), five studies focused on depressive symptoms as an outcome (see Table 2). Two of these studies also had other outcomes.

**Table 2:** Symptoms of MHP in childhood, bullying, and depression as an outcome

NR	Reference	Year	Initial age	Outcome age	Symptoms of MHP in childhood	Higher risk of depressive symptoms	Quality^a^
#1	31	2012	8	13	Irritability, ODD symptoms	YES	+ +
#3	32	2015	5	13	Dysregulation, emotion aggression	YES	+
					Internalising problems	NO	
#7	33	2012	10	12	Depression	NO	–
#9	34	2016	8	29	Behavioural and emotional problems	YES	++
#11	35	2015	5	12	Externalising problems	YES	++

NR refers to the number in the reference list of studies used

^a^CASP, high quality is shown by ++ (> 17 points), medium as + and low as—(< 12 points)

Four studies used different measures of externalising symptoms in childhood, such as irritability [31], aggression [32] or emotional and behavioural problems [32–34]. All studies confirmed a relation to later depressive symptoms following bullying victimization. In the two studies [32, 35] that used measures of internalising symptoms in childhood, no such association was found. Children with irritability or aggression were at risk of being disliked (i.e., peer rejection) [32] and bullied [31, 32, 34], and this increased the risk for later depressive symptoms. One study [32] measured both internalising and externalising symptoms in childhood and found that while emotional dysregulation made a unique contribution to later depressive symptoms following bullying victimisation, the effect from externalising symptoms in childhood to later internalising symptoms mediated by bullying was small. The link between internalising symptoms in childhood and additional depressive symptoms was not mediated by bullying in two studies that measured internalizing symptoms in childhood [32, 35]. The findings thus support a path from externalising symptoms in childhood to internalising symptoms in adolescence via bullying exposure. However, no support was found for internalizing symptoms in childhood leading to additional depressive symptoms in adolescence via bullying exposure.

In addition, five studies measured early symptoms using a different outcome of MHP other than depressive symptoms (see Table 3).

**Table 3:** Symptoms of MHP in childhood, bullying, and additional symptoms of mental health as an outcome

NR	Reference	Year	Initial age	Outcome age	Symptoms of MHP in childhood	Higher risk of MHP	Quality^a^
#3	32	2015	5	13	Dysregulation of emotion and aggression Internalising problems	NO	+
#4	36	2012	5	12	Behavioural- and emotional problems	YES	+
#6	37	2009	8	25	Depression Norm-breaking behaviour	YES	++
#9	34	2016	8	29	Behavioural and emotional problems	YES	++
#10	38	2007	8	18	Behavioural and emotional problems	YES	++

NR refers to the number in the reference list

^a^CASP, high quality is shown by ++ (> 17 points), medium as + and low as—(< 12 points)

All studies in Table 3 used externalising symptoms in childhood as initial measurement, and some studies also included a measurement of internalising symptoms in childhood [32, 36]. In terms of outcome measures, two studies focused on self-harm, either with suicidal intent [36] or without suicidal intent [37], two studies had a more overarching category of psychiatric treatment [33, 38], and one study focused on externalizing problems, specifically delinquency and school difficulties [32].

Four out of the five studies in Table 3 supported a relationship between initial symptoms of externalising behaviours, bullying victimisation, and later MHP [33, 36–38]. Three studies found a relation between early symptoms of behavioural and emotional problems and later MHP [33, 36, 38]. These studies used medical records as outcomes regarding suicide and suicide attempts [36] or all types of psychiatric diagnosis [33, 38].

One study [32] found an association between emotional dysregulation via victimisation and depression (see Table 2) but did not find any connection to externalising outcomes such as school adjustment difficulties or delinquent behaviours. Here aggression and dysregulation appeared to be related via peer rejection to these outcomes, but not via bullying victimisation.

In summary, children with symptoms, such as behavioural problems, had a higher risk of being bullied and having MHP in adolescence, especially internalising mental health issues.

### Children with Diagnosed Disorders

Longitudinal studies focusing on children with diagnosed disorders, rather than on children with symptoms in childhood, and the relationship between bullying victimisation and later MHP, were fewer (k = 5). All identified studies focused on specific neurodevelopmental disorders. The follow-up period tended to be shorter than for the studies that measured symptoms of MHP in childhood. Of the five studies on diagnosed disorders, no study had a follow-up period longer than 5 years, and two studies only had a 2 year follow-up period [39, 40]. In one study, follow-up was when the children were only 6 years old [40]. Four studies had high quality and one had medium quality.

The measure of disability used in these studies was identified diagnoses through either clinical interviews or medical records. Four studies compared children with and without disabilities [37, 40–42]. One study compared those with developmental coordination disorder (DCD) who were bullied vs those who were not [39] (see Table 4).

**Table 4:** Children with diagnosed disorders, bullying, and MHP as an outcome

NR	Reference	Year	Initial age	Age at outcome	Disorder	Higher risk of MHP	Quality^a^
#2	39	2012	7	12	Autistic traits	NO	++
#4	36	2012	5	12	ADHD, ODD borderline	YES	+
#5	40	2017	7	12	ADHD	YES	++
#8	41	2012	7	9–10	DCD	YES	++
#12	42	2013	4	6	ADHD	NO	++

NR refers to the number in the reference list

^a^CASP, high quality is shown by +  + (> 17 points), medium as + and low as—(< 12 points)

Three studies confirmed a relationship between disability, bullying, and later MHP [37, 39, 42]. These studies used different outcome measures of internalising symptoms: psychotic episodes [42], self-harm [37], Total difficulties using SDQ [39]. Two studies did not identify a relationship between disability, bullying, and later MHP [40, 41]. These two studies also measured internalising symptoms as an outcome: psychotic traits [33], anxiety [36] making the results of these five studies that used a diagnosis-based approach inconclusive. There is a lack of studies focusing on diagnosed disorders and later depressive symptoms, none of these studies had a follow-up time extending into adolescence.

## Discussion

In general, the combined results support our hypothesis that children with disabilities will have a higher risk of developing MHP as adolescents if they have been bullied compared to children who have not been bullied or only have a disability. However, two aspects need to be considered: the results are equivocal when only considering children with diagnosed disorders; only 3 out of 5 studies supported our hypothesis. In addition, regarding diagnosed disorders, only studies focusing on neurodevelopmental disorders were found. Second, the only relationships identified concerned externalising symptoms in childhood and internalising symptoms as outcomes, especially depressive symptoms. Regarding studies including children with diagnosed disorders, no study focused on depression specifically as an outcome. The outcomes used were other than for those used in studies that measured symptoms of MHP in childhood.

The studies that measured symptoms early in life primarily focused on outward, behavioural aspects, i.e., emotional dysregulation, norm-breaking behaviour, aggression, and behavioural problems. Difficulties with self-regulation are common in many neurodevelopmental disorders as well; the two population groups (children with symptoms or disorders) might overlap in this way. Children with ADHD are present in several studies [37, 40, 42], and one study focused on DCD [39]. Of these, all but one [40] found a relation between disorder and MHP as outcome mediated by bullying. The study that did not support this relation only had a 2 year follow-up and focused on 4- to 6-year-olds. This aberrant finding might be explained by the fact that MHP are more prevalent in adolescents [26].

Often children with symptoms of MHP or neurodevelopmental disorders have difficulties in the social context and have less access to protective factors, such as good social abilities. Children with higher levels of social skills had lower anxiety at follow-up [42]. Additionally, two other studies found that behaving aggressively in different forms toward peers might lead to peer rejection [31, 32]. Even though peer rejection might not always increase the likelihood of being bullied, it indicates that children with early difficulties may find themselves excluded from social situations. Such situations are essential for positive development as they provide environments for children to learn and improve their social abilities. Therefore, as these issues are visible early in life, it is vital to support these children’s management of their social context. While disorders are not always preventable, adaptations and support from the environment could lead to less problematic behaviour, which could reduce the risk of other negative experiences and thus also reduce the risk of later mental health problems.

Children’s behaviour will impact their surroundings through a reciprocal interaction [8]. Children who have a higher risk of developing depression tend to display early externalising behaviours, such as defiance [31], aggression [32], or behavioural problems [37]. The display of these externalising behaviours increases the likelihood of being disliked, rejected [32], or chosen as victims of bullying, perhaps for not having as many friends to protect them [43, 44]. Being rejected might increase negative behaviours, and over time, these children who externalise problems may also develop internalising issues, such as depression. The two studies on a Finnish sample [33, 38] included more than just internalising issues, such as depression, in their psychiatric disorder outcome. However, they supported the notion that early behavioural problems will increase the likelihood of less than optimal social and mental development. Children who poorly manage their contextual demands will risk peer victimisation and later psychiatric disorders.

Based on a biopsychosocial model [20], MHP in childhood produce a more conclusive finding than focusing on the diagnosed disorder as a measure of disability. This finding can be supported by the view that the role of environmental, contextual factors interacting with outward displays of symptoms serves to create disability [20]. The results indicate that having externalising behaviours in childhood is a risk for unnecessary, additional MHP in adolescence due to being bullied. The studies that measured symptoms early in life primarily focused on behavioural aspects, i.e., emotional dysregulation, norm-breaking behaviour, aggression, and behavioural problems. Many children with neurodevelopmental disorders also display these behavioural aspects, but they do not necessarily need to. The diagnosis does not give information regarding the contextual adaptations made due to the disorder nor the in-group variance of symptoms. Therefore, a categorical definition of diagnosed disorders does not give us information about barriers and facilitators the way symptoms might. This lack of contextual information could explain the inconclusive results for those with diagnosed disorders. However, symptoms of MHP, arguably, indicate a mismatch between a person’s ability and contextual demands. Adding risk factors for MHP in adolescence, in this case, bullying, the risk for additional internalising symptoms increases [44]. Having a neurodevelopmental disorder increases the likelihood of environmental barriers, raising awareness of the need for adaptations. It could be argued that behavioural problems, rather than a diagnosis, are of more relevance in the developmental relation between bullying and MHP. What weakens this argument is that we do not have any studies that look at children who have similar diagnosed disorders of other sensory or physical impairments.

Measurement of internalising outcomes was not the same between those with childhood symptoms of MHP and those with a diagnosed disorder. No identified study focused on depressive symptoms alone for children with neurodevelopmental disorders. At the same time, this was the most common outcome in studies of children with childhood symptoms of MHP. Previous research has shown a strong link between internalising symptoms and bullying, and the most common measure of this is depressive symptoms [21].

From a systems theory perspective, bullying is a significant environmental trauma [2] and is a barrier to participation in meaningful life situations. Many children with disabilities have reduced participation in everyday activities, either in the number of activities [45, 46] or level of intensity [46]. The degree of participation outside of school seems to vary in relation to different aspects of the environment rather than the type and severity of impairment [47]. Many children with disabilities have sedentary activities in the home, such as watching TV [48]. Children who are bullied watch much more TV than children who are not bullied, and children with disorders who are bullied even more so. Also, girls with ADHD have a high risk of peer rejection, predicting poor social adjustment [49]. Sedentary home activities can be one way of handling social isolation, especially concerning social skills or self-regulation difficulties. Over time, this could explain low participation in peer activities, less social adjustment, and increased risk of continued internalising problems [19].

### Strengths and Limitations

This study is a limited systematic review; grey literature was not included, and not all parts of the screening were independently conducted by two reviewers. Despite our effort to identify previous research on the longitudinal association between disability/early symptoms of MHP, bullying, and later MHP, only 12 studies were found. These studies were heterogeneous concerning the type of participants, symptoms of MHP in childhood and diagnosed disorders, length of follow-up, and type of outcomes. A variety of internalising and externalising problems were identified. Having two groups of participants can create conceptual confusion concerning what is related to impairment and health conditions and what is related to environmental barriers. The notion that results seem to be related to behaviours visible in the context, such as externalising behaviours childhood or ADHD rather than internalising symptoms in childhood, provide support for a symptoms-based approach. However, the lack of studies that investigated depression as an outcome for children with diagnosed disorders is also a weakness. Neurodevelopmental disorders can increase the risk of mental health problems, especially emotional symptoms [19]. In a cross-sectional study, children with ADHD had stronger associations with MHP and being bullied [50]. However, longitudinal studies of adolescents support a relation between ASD [51] and ADHD [44, 52] and later depression mediated through bullying. Arguably, the results of the current study point to a convincing conclusion that having two risk factors, such as a disability and being bullied, increases the risk for later MHP.

The identified studies had different lengths of follow-up, which may affect the results. The studies with the most extended follow-up gave the most substantial support for our hypothesis [27, 30, 31]. In studies of adolescents, emotional symptoms may be more stable over time than bullying victimisation, which reduces the predictability of bullying victimisation over time [53]. Perhaps earlier MHP have more time to affect development than later symptoms, which would fit with the developmental, transactional theory [5]. The studies including diagnosed disorders all had short follow-ups, which could be an additional reason for the inconclusiveness. There is thus a need for further longitudinal studies on children with disabilities, their mental health, and traumatic experiences such as peer victimisation. Moreover, studies need to start pre-adolescence to capture the beginning of the process of proximal negative development. Having a disability is a risk factor for poor mental health and being bullied, as the current study found, together creates even worse prospects for these children. Hence, it is vital to gain further knowledge of these early processes.

Another limitation is that dual-blinded coding was not conducted for all full-text papers due to a lack of resources. However, a close collaboration existed throughout the coding process, and all dual coding was conducted independently. A third limitation might be that we separated the studies using a more bio-psycho-social measure of disability in line with the ICF definition, i.e., those that have early symptoms. This group might contain children with medical diagnoses, not (yet) measured by researchers. However, it is likely that these children will later receive a diagnosis. Separating the presentation of results by those with a diagnosis based on a medical model and those with a disability from a more bio-psycho-social perspective was due to differences in inclusion criteria and baseline measurements. This way of presenting the results also allowed exploration of whether a medical diagnosis mattered more than symptoms.

All identified studies related to behaviour visible in context rather than as a bodily visibility. There is a lack of longitudinal studies focusing on physical disabilities or on other types of chronic conditions related to bullying and MHP. Because children with these disabilities are often diagnosed early, future research could focus on following their developmental trajectories from earlier starting points. We cannot assume that the transactional process will be similar between children with neurodevelopmental disorders or behavioural and emotional dysregulations and those with impairments that affect functioning in other ways. However, having these disabilities does increase the risk of being victimized [5, 17]. Children and adolescents with cerebral palsy do have an increased risk of having MHP [54], even though cerebral palsy is a neurodevelopmental disorder affecting movement is not a physical disability. Arguably, more studies focusing on the relationship between sensory, physical, or intellectual disabilities and their relationship with bullying and mental health are needed to generalize findings to other kinds of body impairment, activity limitations, and participation restrictions than were covered by the current review.

## Summary

Many school-aged children experience bullying, and those with disabilities are more likely to be victimized [5–7, 32, 52]. Being bullied increases the likelihood of later internalising symptoms, such as depression. Whilst there are many studies on bullying and MHP, these are primarily cross-sectional studies [21]. The current review only identified 12 studies with a longitudinal design that examined the developmental paths for children with a diagnosis/early symptoms of MHP exposed to bullying and their later MHP. These 12 studies primarily focused on childhood psychological symptoms or neurodevelopmental disorders. We found no longitudinal studies that included children with learning disabilities, intellectual disabilities, or physical disabilities, even though these groups also have an increased risk of being bullied [7]. Cross-sectional or retrospective studies usually find higher correlations between MHP and bullying for typically developing children and children with disabilities [47, 48, 54]. Arsenault [6] argued that children with externalising behaviour are more likely to be bullied and have poorer mental health outcomes. The current review’s results support the finding that early externalising problems were more closely related to later internalising issues via exposure to bullying. However, the sub-set of studies that used a confirmed diagnosis at baseline did not support a clear relationship between bullying and later MHP. This inconclusiveness might indicate that the externalising behaviours have a more significant impact on the transactional process than a diagnosis does. A diagnosis does not automatically indicate an issue with social interaction or lack of appropriate adaptations,externalising behaviours, visible in the social context, indicate a mismatch between expectations and adaptations. Alternatively, measuring symptoms rather than diagnoses results in broader inclusion criteria in studies and reduces bias due to design, which captures more significant amounts of variance, i.e., changes in MHP following bullying.

## Appendix

See Tables 5 and 6.

**Table 5:** Brief description of included studies

Nr	Study	Initial age	N	Age at outcome	Nr of waves	Early symptom or diagnosis	Outcomes	Results	Quality
#1	Barker et al. [31]	8 years	5923	13 years	3	Irritability ODD symptoms	Depression callousness	Children with a high degree of irritability have an increased risk of bullying and increased risk of internalising symptoms. However, there was no relation to callousness	++
#2	Bevan et al. [41]	7 years	6439	12 years	3	Autistic traits 3 years and 7 years	Psychotic traits	There was no mediation between peer victimisation and later psychotic tra. Some childhood autistic traits are related to later psychotic experiences such as odd rituals and unusual habits. The association between the number of autistic traits and psychotic experiences did not change when adjusting for peer victimisation	++
#3	Bierman et al. [32]	5 years	623	12 years	7	Dysregulation of emotion and aggression	Depression	Dysregulation was linked to bullying and bullying to depression and social difficulties Depression in 7th grade was predicted directly through internalising and dysregulation through bullying. Aggression and dysregulation were directly linked to being disliked but disliked were not related to bullying or depression	+
							School difficulty	Aggression and dysregulation were directly linked to school difficulties and peer rejections. Aggression was not related to victimization and victimization was not related to school difficulties	
							Delinquency	Aggression was related to delinquency and both dysregulation and aggression were related to peer rejection. Peer rejection mediated the relation between dysregulation and aggression on delinquency and school difficulties	
#4	Fisher et al. [37]	5 years	2141	11 years	4	Behavioural problems ADHD, ODD Borderline	Self-harming	Bullied children are at the highest risk of self-harming. Only 2.9% (62) children self-harmed of these 35 or 56% had experienced bullying Exposure to frequent bullying predicted higher rates of self-harm even after children’s pre-morbid emotional and behavioural problems, low IQ, and family environmental risks were considered The risk factors for self-harm in bullied children were much higher for children with ADHD and conduct and extreme borderline characteristics	+
# 5	Hennig et al. [42]	7 years	8241 80 Dis	5 years	3	ADHD combined subtype C	Psychotic episodes	Children with ADHD combined subtype C had a threefold risk for psychotic experiences and to be involved in bullying. Those only involved in bullying had a two-fold risk for psychotic experiences at 12.8 years	++
#6	Klomek et al. [36]	8 years	5813	up to 16 years	2	Depression and norm-breaking behaviour	Suicide and attempted suicide	Conduct and depression were measured at 8 years by parents, childhood bullying at 8 years by teachers and later suicide and suicide attempts up to 29 years. The study indicated that boys frequently victimised had an increased risk and that this risk was even higher for those with initial depression. For boys, conduct reduced the risk for later suicide or attempt Girls showed a similar pattern as boys but with lower risk than boys There is an increased risk for those with both risk factors. However, bullying had a more significant impact	++
#7	Kochel et al. [35]	10 years	486	2 years	3	Depression T1	Depression	Depression was stable from 10 to 12 years. Being depressed increased the likelihood of being bullied; however, being bullied did not increase the likelihood of depression	–
#8	Lingham et al. [39]	7 years	6902 FH = 235	2 years	3	DCD	Total difficulty	Children with DCD who were bullied, rated by parents, had higher risk MHP measured with Total difficulties SDQ	++
#9	Sourander et [33]	8 years	5034	21 years	2	Behavioural and emotional problems 8 years 85th percentile	Psychiatric diagnosis Depression	Two risk factors increased the risk of later psychiatric diagnosis and depression up to 29 years. Of those screened positive for psychiatric symptoms at 8 and who were not bullied, the risk of any psychiatric diagnosis had a 50% increased risk, while those victimised, those with both risk factors had almost three times as high risk	++
#10	Sourander et al. [38]	8 year	2540	18 years	2	Behavioural and emotional problems 8 years 85^th^ percentile	Psychiatric diagnosis	For boys, having early symptoms doubled the risk for later psychiatric problems, but having both risk factors increased the risk 3.5 times	++
#11	Wertz et al. [34]	5 years	2232	12 years	4	Externalising issues at 5 years of age	Internalising behaviour	Bullying victimization mediated the relation between externalising symptoms at 5 years and internalising symptoms at 12 years. Externalising issues also had a direct effect on later internalising behaviours The mediating variable accounted for some but not all the phenotypic associations. Including peers, victimisation increased the impact but did not reduce the effect of externalising problems	++
#12	Wichstrøm et al. [40]	4 years	797	2 years	2	Behavioural inhibition ADHD ODD CD	Anxiety	Inhibition had an indirect link through bullying to future anxiety. ADHD, however, did not; ADHD related to social skills and later anxiety, and it also had a direct link to later anxiety There was a link between bullying and anxiety, which did not change when adjusting for the initial anxiety level. ADHD was related to later anxiety	++

**Table 6:** Search strings used in different databases

Databases	Search strings
Cinahl	( (MH "Stress, Psychological + ") OR ( ( (MH "Disruptive Behavior") OR (MH "Substance Abuse") OR (MH "Self-Injurious Behavior") OR (MH "Anxiety") OR (MH "Depression") OR (MH "Mental Health") OR (MH "Affective Symptoms") OR (MH "Compulsive Behavior") OR (MH "Suicide + ")) OR AB ( mental health or mental illness or mental disorder or psychiatric illness) OR AB psychosomatic OR AB behavior problems OR AB ( addiction or substance abuse or drug abuse) OR AB depression OR AB anxiety OR AB suicide OR AB compulsive behavior OR AB conduct) OR AB ( stress or psychological stress or emotional stress)) AND ( ( (MH "Cyberbullying") OR (MH "Bullying + ")) OR AB ( victimiz* OR bully* or cyberbully*)) AND ( ( MH disability OR AB ( disability or disabilities or disabled)) OR (MH "Chronic Disease + ") AND AB ( chronic illness or chronic disease)) AND ( MH child OR MH ( adolescen' or juvenil' or teen' or youth or young person or young people) OR MH students OR AB ( children or kids or youth or child) OR AB ( adolescents or teenagers or young adults))
Eric	( DE "Late Adolescents" OR DE "Adolescents" OR DE "Youth" OR DE "Middle School Students" OR DE "Early Adolescents" OR DE "Elementary School Students" OR DE "High School Students" OR DE "Junior High School Students" OR DE "Preadolescents") AND ( ( DE "Disabilities" OR DE "Adventitious Impairments" OR DE "Attention Deficit Disorders" OR DE "Behavior Disorders" OR DE "Communication Disorders" OR DE "Congenital Impairments" OR DE "Developmental Disabilities" OR DE "Diseases" OR DE "Hearing Impairments" OR DE "Injuries" OR DE "Intellectual Disability" OR DE "Language Impairments" OR DE "Learning Disabilities" OR DE "Mental Disorders" OR DE "Mild Disabilities" OR DE "Multiple Disabilities" OR DE "Perceptual Impairments" OR DE "Physical Disabilities" OR DE "Severe Disabilities" OR DE "Special Health Problems" OR DE "Speech Impairments" OR DE "Visual Impairments" OR DE "Developmental Delays" OR DE "Gifted Disabled") OR AB disability OR AB disord* OR AB chronic illness OR AB chronic disease) AND ( ( (((DE "Behavior Disorders" OR DE "Self Destructive Behavior") OR (DE "Mental Health")) OR (DE "Behavior Problems" OR DE "Depression (Psychology)" OR DE "Emotional Problems" OR DE "Psychosomatic Disorders" OR DE "Emotional Disturbances" OR DE "Psychopathology")) OR (DE "Anxiety")) OR DE suicide OR DE stress) AND ( ( (AB bully*) OR ( DE "Bullying")) OR AB victimization OR (AB "cyberbully*"))
Medline	( ( (MH "Stress, Psychological + ") OR ( ( (MH "Disruptive Behavior") OR (MH "Substance Abuse") OR (MH "Self-Injurious Behavior") OR (MH "Anxiety") OR (MH "Depression") OR (MH "Mental Health") OR (MH "Affective Symptoms") OR (MH "Compulsive Behavior") OR (MH "Suicide + ")) OR AB ( mental health or mental illness or mental disorder or psychiatric illness) OR AB psychosomatic OR AB behavior problems OR AB ( addiction or substance abuse or drug abuse) OR AB depression OR AB anxiety OR AB suicide OR AB compulsive behavior OR AB conduct) OR AB ( stress or psychological stress or emotional stress)) AND ( ( MH disability OR AB ( disability or disabilities or disabled)) OR (MH "Chronic Disease + ") AND AB ( chronic illness or chronic disease)) AND ( ( (MH "Cyberbullying") OR (MH "Bullying + ")) OR AB ( victimiz* OR bully* or cyberbully*)) AND ( MH child OR MH ( adolescen' or juvenil' or teen' or youth or young person or young people) OR MH students OR AB ( children or kids or youth or child) OR AB ( adolescents or teenagers or young adults)))
Pubmed	(((((((""Bullying""[Mesh]) OR ""Cyberbullying""[Mesh])))) AND (((((((((((((""Disabled Children""[Mesh]) OR ""Mental Disorders""[Mesh]) OR ""Congenital, Hereditary, and Neonatal Diseases and Abnormalities""[Mesh]) OR ""Disorders of Environmental Origin""[Mesh]) OR ""Musculoskeletal Diseases""[Mesh]) OR ""Nervous System Diseases""[Mesh]) OR ""Developmental Disabilities""[Mesh]) OR ""Neurodevelopmental Disorders""[Mesh]) OR ""Communication Disorders""[Mesh]) OR ""Learning Disorders""[Mesh]) OR ""Chronic Disease"" [Mesh])))) AND ((((""Child""[Mesh]) OR ""Child, Preschool""[Mesh]) OR ""Adolescent""[Mesh]))) AND ((((((""Behavioral Symptoms""[Mesh]) OR ""Mental Health""[Mesh]) OR ""Behavior, Addictive""[Mesh]) OR ""Mental Disorders""[Mesh]) OR ""Stress, Psychological""[Mesh]))
Psychinfo	(MAINSUBJECT.EXACT.EXPLODE("Cyberbullying") OR MAINSUBJECT.EXACT.EXPLODE("Bullying") OR MAINSUBJECT.EXACT.EXPLODE("victimization")) AND (MAINSUBJECT.EXACT.EXPLODE("Disabilities") OR MAINSUBJECT.EXACT("Diagnosis") OR MAINSUBJECT.EXACT.EXPLODE("Syndromes") OR MAINSUBJECT.EXACT.EXPLODE("Disorders") OR MAINSUBJECT.EXACT.EXPLODE("Chronic Illness")) AND ((noft(MAINSUBJECT.EXACT.EXPLODE) (noft("Psychological Stress"))) OR (MAINSUBJECT.EXACT("Child hood Play Behavior") OR MAINSUBJECT.EXACT("Eating Behavior") OR MAINSUBJECT.EXACT("Social Behavior") OR MAINSUBJECT.EXACT("Self-Injurious Behavior") OR MAINSUBJECT.EXACT("Health Behavior") OR MAINSUBJECT.EXACT("Lifestyle") OR MAINSUBJECT.EXACT("Obscenity") OR MAINSUBJECT.EXACT("Drinking Behavior") OR MAINSUBJECT.EXACT("Conduct Disorder") OR MAINSUBJECT.EXACT("Classroom Behavior") OR MAINSUBJECT.EXACT.EXPLODE("Addiction") OR MAINSUBJECT.EXACT("Behavior Problems") OR MAINSUBJECT.EXACT("Coping Behavior") OR MAINSUBJECT.EXACT.EXPLODE("Emotional Style") OR MAINSUBJECT.EXACT("Anger") OR MAINSUBJECT.EXACT("Grief") OR MAINSUBJECT.EXACT.EXPLODE("Emotional States")))Limits applied Databases: Limited by: Age group: Adolescence (13–17 Yrs), childhood (birth-12 Yrs), Preschool Age (2–5 Yrs), School Age (6–12 Yrs)
Scopus	( ( TITLE-ABS-KEY ( disab*) OR TITLE-ABS-KEY ( "chronic condition" *) OR TITLE-ABS-KEY ( disorder*)) AND ( TITLE-ABS-KEY ( bully*) OR TITLE-ABS-KEY ( cyberbully*) OR TITLE-ABS-KEY ( victimiz*)) AND ( TITLE-ABS-KEY ( child*) OR TITLE-ABS-KEY ( adolescen*) OR TITLE-ABS-KEY ( student) OR TITLE-ABS-KEY ( pupil)) AND ( TITLE-ABS-KEY ( mental AND health) OR TITLE-ABS-KEY ( stress) OR TITLE-ABS-KEY ( psychosomatic AND complaints) OR TITLE-ABS-KEY ( depression) OR TITLE-ABS-KEY ( anxiety) OR TITLE-ABS-KEY ( addiction) OR TITLE-ABS-KEY ( self-injury) OR TITLE-ABS-KEY ( conduct) OR TITLE-ABS-KEY ( hyper-activity) OR TITLE-ABS-KEY ( "Behavioral problem") OR TITLE-ABS-KEY ( emotional AND problem)))
Sociological abstracts	(MAINSUBJECT.EXACT("Aggression") OR noft(bullying) AND (MAINSUBJECT.EXACT.EXPLODE("Adolescents") OR MAINSUBJECT.EXACT.EXPLODE("Children"))) AND (MAINSUBJECT.EXACT.EXPLODE("Physically Handicapped") OR MAINSUBJECT.EXACT.EXPLODE("Disorders") OR MAINSUBJECT.EXACT.EXPLODE("Developmental Disabilities") OR MAINSUBJECT.EXACT.EXPLODE("Congenitally Handicapped") OR MAINSUBJECT.EXACT.EXPLODE("Learning Disabilities") OR MAINSUBJECT.EXACT.EXPLODE("Handicapped")) AND (MAINSUBJECT.EXACT.EXPLODE("Self Destructive Behavior") OR MAINSUBJECT.EXACT.EXPLODE("Mental Illness") OR MAINSUBJECT.EXACT.EXPLODE("Addiction") OR MAINSUBJECT.EXACT.EXPLODE("Emotions") OR MAINSUBJECT.EXACT.EXPLODE("Psychological Stress") OR MAINSUBJECT.EXACT.EXPLODE("Affective Illness") OR MAINSUBJECT.EXACT.EXPLODE("Mental Health") OR MAINSUBJECT.EXACT.EXPLODE("Stress") OR MAINSUBJECT.EXACT.EXPLODE("Psychological Distress") OR MAINSUBJECT.EXACT.EXPLODE("Behavior"))
Svepub	Bullying AND disabil* OR Victimiz* and disabil*
Open grey	Bully* AND disab* OR Cyberbully* and Disabil* OR Victimiz AND disabil*
OECD library	(All Fields contains ‘bullying’) from (Language contains ‘en’) **OR** from (All Fields contains ‘victimisation’) **AND** from (All Fields contains ‘child* OR adolescent* OR student*’) **AND** from (All Fields contains ‘Disabilit* OR disorder’) **AND** from (All Fields contains ‘Emotion* OR Behaviour OR depression OR anxiety OR conduct OR hyper OR addict* OR suicide* OR self-harm’) **AND** from (IGO collection contains ‘OECD’)

## References

[1] Bowser J, Larson J, Bellmore A, Olson C (2018). Resnik F (2018) Bullying victimization type and feeling unsafe in middle school. J Sch Nurs.

[2] Silberg JL, Copeland W, Linker J, Moore AA, Roberson-Nay R, York TP (2016). Psychiatric outcomes of bullying victimization: a study of discordant monozygotic twins. Psychol Med.

[3] Awiria O, Olweus D, Byrne B (1994). Bullying at school-what we know and what we can do. Br J Educ Stud.

[4] Olweus D (1994). Bullying at school: basic facts and effects of a school based intervention P\program. J Child Psychol Psychiatry.

[5] Rose CA, Monda-Amaya LE, Espelage DL (2011). Bullying perpetration and V\victimization in special education: a review of the literature. Remedial Spec Educ.

[6] Arseneault L, Bowes L (2010). Shakoor S (2010) Bullying victimization in youths and mental health problems: “Much ado about nothing”?. Psychol Med.

[7] Pinquart M (2017). Systematic review: bullying involvement of children with and without chronic physical illness and/or physical/sensory disability-a aeta-analytic comparison with healthy/nondisabled peers. J Pediatr Psychol.

[8] Sameroff AJ, Fiese BH, Shonkoff JP, Meisels SJ (2000). Transactional regulation: the developmental ecology of early intervention. Handbook of early childhood intervention.

[9] Bronfenbrenner U, Morris PA, Damon W, Lerner RM (2007). The bioecological model of human development. Handbook of child psychology.

[10] Bronfenbrenner U, Morris PA, Damon W, Lerner RM (1998). The ecology of developmental processes. Handbook of child psychology theoretical models of human development.

[11] Bowes L, Maughan B, Ball H, Shakoor S, Ouellet-Morin I, Caspi A, Moffitt TE, Arseneault L (2013). Chronic bullying victimization across school transitions: the role of genetic and environmental influences. Dev Psychopathol.

[12] Arseneault L, Walsh E, Trzesniewski K, Newcombe R, Caspi A, Moffitt TE (2006). Bullying victimization uniquely contributes to adjustment problems in young children: a nationally representative cohort study. Pediatrics.

[13] Houtepen LC, Heron J, Suderman MJ, Fraser A, Chittleborough CR, Howe LD (2020). Associations of adverse childhood experiences with educational attainment and adolescent health and the role of family and socioeconomic factors: a prospective cohort study in the UK. PLoS Med.

[14] Kumpulainen K, Räsänen E (2000). Children involved in bullying at elementary school age: their psychiatric symptoms and deviance in adolescence: an epidemiological sample. Child Abuse Negl.

[15] Takizawa R, Maughan B, Arseneault L (2014). Adult health outcomes of childhood bullying victimization: evidence from a five-decade longitudinal British birth cohort. Am J Psychiatry.

[16] Kim YS, Leventhal BL, Koh Y-J, Hubbard A, Boyce WT (2006). School bullying and youth violence: causes or consequences of psychopathologic behavior?. Arch Gen Psychiatry.

[17] Sansone RA, Watts DA, Wiederman MW (2014). Being bullied in childhood, and pain and pain perception in adulthood. Int J Soc Psychiatry.

[18] WHO (1948). Constitution of the World Health Organization New York: World Health Organization.

[19] Granlund M, Imms C, King G, Andersson AK, Augustine L, Brooks R, Danielsson H, Gothilander J, Ivarsson M, Lundqvist L-O, Lygnegård F, Almqvist L (2021). Definitions and operationalization of mental health problems, wellbeing and participation constructs in children with NDD: distinctions and clarifications. Int J Environ.

[20] WHO (2001). International classification of functioning, disability, and health: ICF.

[21] Guzman‐Holst C, Bowes L, Smith PK, O’Higgins Norman J (2021). Bullying and internalizing symptoms. Wiley-Blackwell handbook of bullying: a comprehensive and international review of research and intervention.

[22] Brendgen M, Comtois-Cabana M, Ouellet-Morin I, Smith PK, O’Higgins Norman J (2021). Genetic and epigenetic factors in bullying. Wiley-Blackwell bullying handbook, characteristics, risks and outcomes.

[23] Veenstra R, Huitsing G, Smith PK, O’Higgins Norman J (2021). Social network. Wiley-Blackwell bullying handbook, characteristics, risks and outcomes.

[24] Maïano C, Aimé A, Salvas M-C, Morin AJS, Normand CL (2016). Prevalence and correlates of bullying perpetration and victimization among school-aged youth with intellectual disabilities: a systematic review. Res Dev Disabil.

[25] Maïano C, Normand CL, Salvas M-C, Moullec G, Aimé A (2016). Prevalence of school bullying among youth with autism spectrum disorders: a systematic review and meta-analysis: school bullying and autism spectrum disorders. Autism Res.

[26] Inchley J, Currie D, Budisavljevic S, Torsheim T, Jåstad A, Cosma A (2020). Spotlight on adolescent health and well-being. Findings from the 2017/2018 Health Behaviour in School-aged Children (HBSC) survey in Europe and Canada. International report. Key data Copenhagen.

[27] Bjereld Y, Augustine L, Thornberg R (2020). Measuring the prevalence of peer bullying victimization: review of studies from Sweden during 1993–2017. Child Youth Serv Rev.

[28] Ouzzani M, Hammady H, Fedorowicz Z, Elmagarmid A (2016). Rayyan-a web and mobile app for systematic reviews. Syst Rev.

[29] Moher D, Liberati A, Tetzlaff J, Altman DG (2009). Preferred reporting items for systematic reviews and meta-Analyses: the PRISMA statement. PLoS Med.

[30] CASP (2018) CASP Cohort study checklist [online]. In https://casp-uk.net/casp-tools-checklists/

[31] Barker ED, Salekin RT (2012). Irritable oppositional defiance and callous unemotional traits: is the association partially explained by peer victimization?. J Child Psychol Psychiatry.

[32] Bierman KL, Kalvin CB, Heinrichs BS (2015). Early childhood precursors and adolescent sequelae of grade school peer rejection and victimization. J Clin Child Adolesc Psychol.

[33] Sourander A, Gyllenberg D, Brunstein Klomek A, Sillanmäki L, Ilola A-M, Kumpulainen K (2016). Association of bullying behavior at 8 years of age and use of specialized services for psychiatric disorders by 29 years of age. JAMA Psychiat.

[34] Wertz J, Zavos H, Matthews T, Harvey K, Hunt A, Pariante CM, Arseneault L (2015). Why some children with externalising problems develop internalising symptoms: testing two pathways in a genetically sensitive cohort study. J Child Psychol Psychiatry.

[35] Kochel KP, Ladd GW, Rudolph KD (2012). Longitudinal associations among youth depressive symptoms, peer victimization, and low peer acceptance: an interpersonal process perspective. Child Dev.

[36] Klomek AB, Sourander A, Niemelä S, Kumpulainen K, Piha J, Tamminen T, Gould MS (2009). Childhood bullying behaviors as a risk for suicide attempts and completed suicides: a population-based birth cohort study. J Am Acad Child Adolesc Psychiatry.

[37] Fisher HL, Moffitt TE, Houts RM, Belsky DW, Arseneault L, Caspi A (2012). Bullying victimisation and risk of self-harm in early adolescence: longitudinal cohort study. BMJ.

[38] Sourander A, Jensen P, Ronning JA, Niemela S, Helenius H, Sillanmaki L, Almqvist F (2007). What is the early adulthood outcome of boys who bully or are bullied in childhood? The Finnish “From a Boy to a Man” study. Pediatrics.

[39] Lingam R, Jongmans MJ, Ellis M, Hunt LP, Golding J, Emond A (2012). Mental health difficulties in children with developmental coordination disorder. Pediatrics.

[40] Wichstrøm L, Belsky J, Berg-Nielsen TS (2013). Preschool predictors of childhood anxiety disorders: a prospective community study. J Child Psychol Psychiatry.

[41] Bevan JR, Thapar A, Lewis G, Zammit S (2012). The association between early autistic traits and psychotic experiences in adolescence. Schizophr Res.

[42] Hennig T, Jaya ES, Lincoln TM (2017). Bullying mediates between Attention-Deficit/Hyperactivity Disorder in childhood and psychotic experiences in early adolescence. Schizophr Bull.

[43] Sigurdson JF, Kaasbøll J, Sund AM, Smith PK, O’Higgins Norman J (2021). Bullying and externalizing. Wiley-Blackwell bullying handbook, characteristics, risks and outcomes.

[44] Roy A, Hartman CA, Veenstra R, Oldehinkel AJ (2015). Peer dislike and victimisation in pathways from ADHD symptoms to depression. Eur Child Adolesc Psychiatry.

[45] Almqvist L (2006) Children’s health and developmental delay: positive functioning in everyday life. Dissertation, Örebro, Örebro university Sweden

[46] Ullenhag A, Krumlinde-Sundholm L, Granlund M, Almqvist L (2014). Differences in patterns of participation in leisure activities in Swedish children with and without disabilities. Disabil.

[47] Mâsse LC, Miller AR, Shen J, Schiariti V, Roxborough L (2013). Patterns of participation across a range of activities among Canadian children with neurodevelopmental disorders and disabilities. Dev Med Child Neurol.

[48] Kremer KP, Kremer TR (2019). Bullying victimization and disability status are associated with television watching in adolescence. J Child Fam Stud.

[49] Kok FM, Groen Y, Fuermaier ABM, Tucha O (2016). Problematic peer functioning in girls with ADHD: a systematic literature review. PLoS ONE.

[50] Holmberg K, HjernK:son Blomquist AH (2009). Health complaints, bullying and predictors of attention-deficit/hyperactivity disorder (ADHD) in 10-year-olds in a Swedish community.

[51] Rai D, Culpin I, Heuvelman H, Magnusson CMK, Carpenter P, Jones HJ, Pearson RM (2018). Association of autistic traits with depression from childhood to age 18 years. JAMA Psychiatry.

[52] Fogleman ND, Leaberry KD, Rosen PJ, Walerius DM, Slaughter KE (2018). Relation between internalizing behaviors, externalizing behaviors, and peer victimization among children with and without ADHD. Atten Defic Hyperact Disord.

[53] Sweeting H, Young R, West P, Der G (2006). Peer victimization and depression in early-mid adolescence: a longitudinal study. Br J Educ Psychol.

[54] Pittet I, Berchtold A, Akré C, Michaud PA, Surís JC (2010). Are adolescents with chronic conditions particularly at risk for bullying?. Arch Dis Child.

